# Supplementary material for: The causal role of circulating inflammatory markers in osteoporosis: a bidirectional Mendelian randomized study
Source: Front Immunol. 2024 Jul 18;15:1412298. doi: 10.3389/fimmu.2024.1412298 (PMC11291241; doi:10.3389/fimmu.2024.1412298)
Supplement: Supplementary file 1 [file DataSheet_1.zip › supplementary material/OSTEOPOROSIS-CIM/GCST90274843.tsv.gz_leave_one_out.pdf]

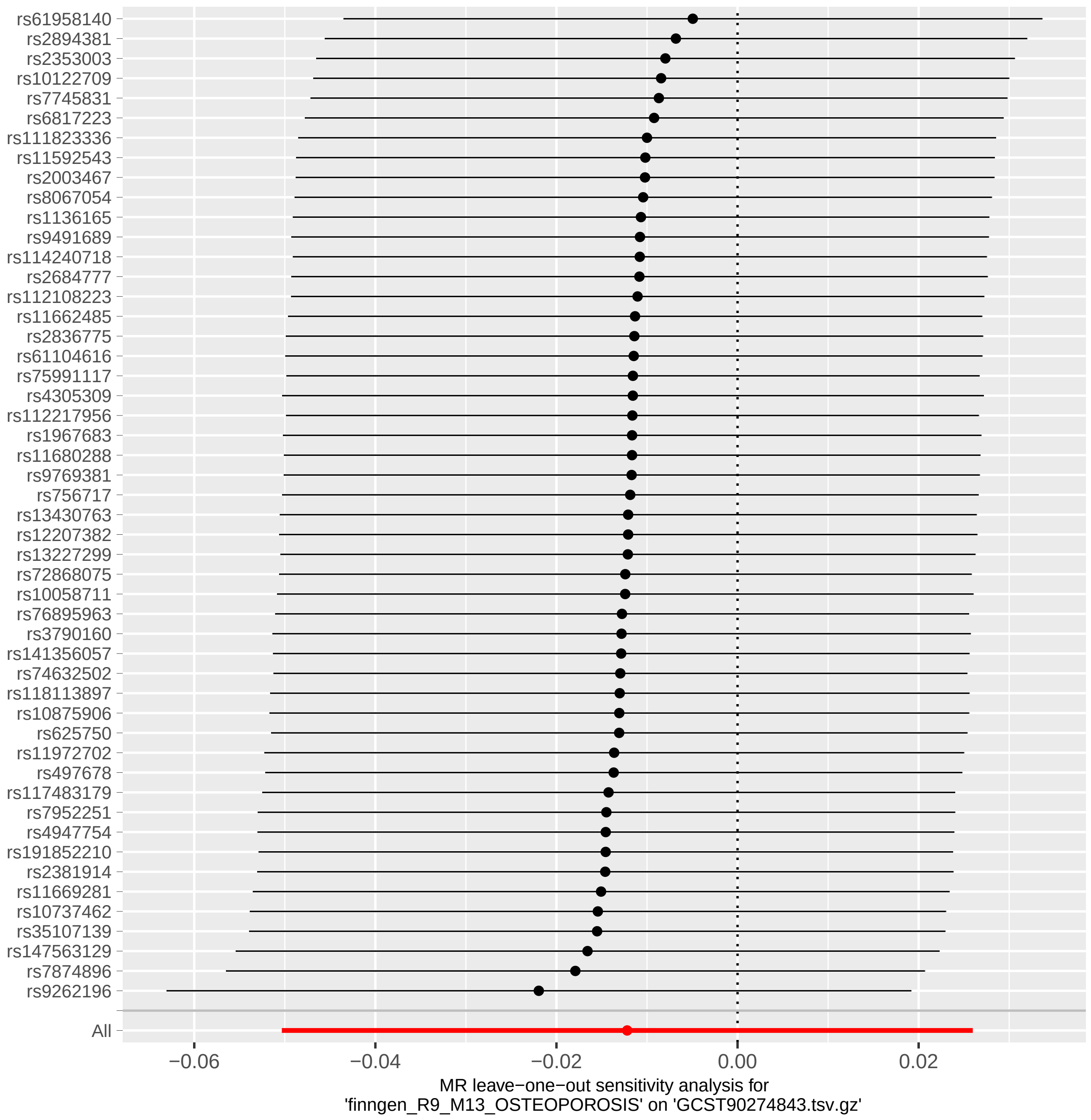
rs61958140
rs2894381
rs2353003
rs10122709
rs7745831
rs6817223
rs111823336
rs11592543
rs2003467
rs8067054
rs1136165
rs9491689
rs114240718
rs2684777
rs112108223
rs11662485
rs2836775
rs61104616
rs75991117
rs4305309
rs112217956
rs1967683
rs11680288
rs9769381
rs756717
rs13430763
rs12207382
rs13227299
rs72868075
rs10058711
rs76895963
rs3790160
rs141356057
rs74632502
rs118113897
rs10875906
rs625750
rs11972702
rs497678
rs117483179
rs7952251
rs4947754
rs191852210
rs2381914
rs11669281
rs10737462
rs35107139
rs147563129
rs7874896
rs9262196
All
−0.06
−0.04
−0.02
0.00
0.02
MR leave−one−out sensitivity analysis for
'finngen_R9_M13_OSTEOPOROSIS' on 'GCST90274843.tsv.gz'
